# Eye care through private sector in Oman

**DOI:** 10.4103/0974-620X.53045

**Published:** 2009

**Authors:** Umashankar, Rajiv Khandekar

**Affiliations:** Welcare Clinic, Muscat, Oman; 1 Eye & Ear Health Care, Ministry of Health

Dear Sir,

Communicable and nutritional eye diseases are declining in Oman while age-related and chronic eye diseases are on the rise.[1] All stakeholders should alter their eye care services accordingly. The primary-secondary eye care complex, in the Ministry of Health (MOH) institutions play a key role in addressing common and blinding eye diseases, and offer services at affordable cost.[2,3] But, family physicians and ophthalmologists of private sector could contribute significantly and perhaps uniquely in looking beyond the goals of VISION 2020, a global initiative launched jointly by the World Health Organization and the International Agency for the Prevention of Blindness.[4]

At present, there are three private hospitals with surgical eye care facilities in Oman. In addition, there are twelve private eye clinics with ophthalmologists, nearly 30 qualified contact lens practitioners, and 150 optical shops.[5] They are registered and approved through the Department of Private Health Establishment, MOH.

The private sector could assist national eye care services and contribute in policy making efforts within VISION 2020 initiative of Oman, in the following key areas:

- Refractive surgery and contact lens practice.
- Corneal transplant and collagen cross-linkage for keratoconus.
- Photo therapy and intravitreal management modalities for retinal diseases such as age-related macular degeneration and advanced cases of diabetic retinopathy.
- Individualized case management of rare eye conditions where multisystem care is needed.
- Low vision care to the adult population.
- Eye screening procedures in private schools for amblyopia and refractive error as per the recommendations of the National policies.
- Offering eye screening for driving licensing, insurance, and preemployment medical checkups.
- Provision of eye care through insurance and payment system to citizen of Oman, thereby reducing the workload of MOH institutions.
- Training Omani mid-level eye care people through collaboration with institutions of international standards.
- Keeping the ophthalmic fraternity knowledgeable through periodic meetings, where internationally renowned guest speakers could be invited.

## References

[1] Khandekar R, Mohammed AJ, Raisi AA (2007). Prevalence and causes of blindness and low vision; before and five years after ′VISION 2020′ initiatives in Oman: a review. Ophthalmic Epidemiol.

[2] Khandekar R, Mohammed AJ (2006). Health Facilities for Primary Eye Care in Sultanate of Oman (Primary Eye Care Study 2000). SQU Journal for Scientific Research: Medical Sciences.

[3] (2000). Ministry of Health. Situation of Eye Health in Eye Health Care Manual.

[4] Resnikoff S, Kocur I, Etya′ale DE, Ukety TO (2008). Vision 2020 - the right to sight. Ann Trop Med Parasitol.

[5] Khandekar R, Fahdi M AI (2009). Evaluation of resources for contact lens practice in private contact lens clinics of Muscat, Oman. Oman J of Ophthal.

